# Antibiotic-Resistant Bacteria in Drinking Water from the Greater Accra Region, Ghana: A Cross-Sectional Study, December 2021–March 2022

**DOI:** 10.3390/ijerph191912300

**Published:** 2022-09-28

**Authors:** Hawa Ahmed, Maria Zolfo, Anita Williams, Jacklyne Ashubwe-Jalemba, Hannock Tweya, Wisdom Adeapena, Appiah-Korang Labi, Lady A. B. Adomako, Gloria N. D. Addico, Regina A. Banu, Mark O. Akrong, Gerard Quarcoo, Selorm Borbor, Mike Y. Osei-Atweneboana

**Affiliations:** 1 Council for Scientific and Industrial Research-Water Research Institute (CSIR-WRI), Achimota, Accra P.O. Box AH 38, Ghana; 2 Institute of Tropical Medicine, 2000 Antwerp, Belgium; 3 MSF Luxembourg Operational Research (LuxOR) Unit, L-1617 Luxembourg, Luxembourg; 4 Medwise Solutions, Nairobi P.O. Box 2356-00202 KNH, Kenya; 5 Malawi International Training and Education Center for Health (Malawi-I-TECH), Lilongwe 3, Lilongwe P.O. Box 30369, Malawi; 6 Kintampo Health Research Centre, Kintampo P.O. Box 200, Bono East, Ghana; 7 WHO Country Office, 7 Ameda Street, Roman Ridge, Accra P.O. Box MB 142, Ghana

**Keywords:** potable water, One Health, antimicrobial resistance (AMR), multidrug resistance, West Africa, Sustainable Development Goal (SDG) 6, SORT IT, operational research

## Abstract

With safely managed water accessible to only 19% of the population in Ghana, the majority of its residents are at risk of drinking contaminated water. Furthermore, this water could be a potential vehicle for the transmission of antimicrobial-resistant bacteria. This study assessed the presence of bacteria and the antibiotic resistance profile of *Escherichia coli* and *Pseudomonas aeruginosa* in drinking-water sources using membrane filtration and Kirby–Bauer disc diffusion methods. A total of 524 water samples were analyzed for total coliforms, total heterotrophic bacteria, *E. coli* and *P. aeruginosa*. Samples included sachets, bottled water, tap water, borehole and well water. Most of the sachet and bottled water samples were within the limits of Ghana’s standards for safe drinking water for the parameters tested. Over 50% of tap and borehole water was also free of *E. coli* and *P. aeruginosa*. Overall, of 115 *E. coli* isolates from tap and ground water samples, most were resistant to cefuroxime (88.7%), trimethoprim–sulfamethoxazole (62.6%) and amoxicillin–clavulanate (52.2%). *P. aeruginosa* isolates were most resistant to aztreonam (48%). Multidrug resistance was predominantly seen among *E. coli* isolates (58%). Evidence from this study calls for routine antimicrobial resistance surveillance in drinking water across the country and additional treatment of water sources at household levels.

## 1. Introduction

Water is core to the survival of humans, animals and plants. Sustainable Development Goal (SDG) 6 calls to “achieve universal and equitable access to safe and affordable drinking water for all by the year 2030” [1]. Globally, 1.8 billion people still use drinking water sources contaminated with fecal matter, and this contamination is more prevalent in Africa [2]. Drinking water is often obtained from surface waters, reservoirs, boreholes or hand-dug wells [3]. In developing countries such as Ghana, inadequate or dysfunctional sewage systems, coupled with waste water treatment plant (WWTP) discharges, and runoff from agricultural lands and animal production systems, further contribute to the contamination of such water sources [4]. In total, 27% of the Ghanaian population have access to tap water, with 29% and 8% resorting to well water and other natural sources, respectively [5]. A national survey indicates that only 19% of the population have access to safely managed drinking-water sources [6].

The wide consumption of antibiotics in humans and animals has favored the development of antibiotic-resistant bacteria (ARB) and antibiotic-resistant genes (ARGs), which are subsequently released through wastewater into the environment, including drinking-water sources [4,7]. Additionally, antimicrobial resistance (AMR) may develop de novo in bacteria under the selective pressure of antimicrobial residues and other chemicals released into water bodies [8]. Ingestion of these resistant bacteria in contaminated drinking water may not only result in infections that have a high risk of treatment failure, but also the possibility of horizontal gene transfer from environmental bacteria to human pathogens [9]. Thus, water may be an important source for the spread of antimicrobial-resistant organisms and genes among humans and animals. 

The problem of AMR is a major health threat with severe consequences, especially for low- and middle-income countries (LMIC) [10,11]. Significant efforts towards tackling this crisis are being made by international organizations such as The World Health Organization (WHO), and the WHO Global Action plan advocates for AMR surveillance as one of its core objectives. However, not much emphasis has been placed on AMR surveillance in water, especially in LMICs such as Ghana. Studies documenting AMR in water are primarily from developed countries, mostly Europe, with a gap in knowledge from LMICs [4]. 

In Ghana, the Council for Scientific and Industrial Research (CSIR)–Water Research Institute (WRI) is mandated to conduct research into all aspect of water resources in order to provide scientific and technological information for the sustainable development, utilization and management of such resources. Analysis of water for domestic consumption and industrial purposes is routinely carried out here. Basic bacteriological tests assessing the quality of drinking water generally look for the absence of “indicator bacteria” such as: (i) total coliforms (TC), fecal coliforms (FC) and *Escherichia coli,* which point to potential fecal contamination, because these organisms are mainly found in the gastrointestinal systems of humans and animals; and (ii) total heterotrophic bacterial (THB) counts which reflect the number of live, culturable heterotrophic bacteria in water [12,13]. Upon request, additional testing for other organisms including *Pseudomonas* spp., *Enterococci* spp. and *Clostridium* spp. are performed. All these tests exclude antimicrobial-susceptibility testing (AST). Antibiotic resistant *E. coli* and *Pseudomonas aeruginosa* have, however, been isolated in drinking water from various sources [14,15,16], and both organisms form part of the WHO Global priority pathogen list for antibiotic resistance [17]. In particular, *P. aeruginosa* may also be isolated from “coliform-free” drinking water sources [18], and is frequently found as antimicrobial-resistant bacteria in health care systems. 

AMR surveillance studies in Ghana have mainly focused on humans and animals, and very little has been reported from the environment [7,19], including the presence of ARB in drinking water sources. There is, therefore, a need for research that bridges this knowledge gap.

This study aimed to assess the presence of bacteria and antibiotic resistance among *E. coli* and *P. aeruginosa* isolates from different water sources from the Greater Accra Region of Ghana, submitted to the CSIR-WRI for analysis between December 2021 and March 2022. This information is instrumental for Ghana’s national AMR action plan and could further bolster interventions to control water contamination. Evidence generated from this study may help advocate for antibiotic-susceptibility testing as part of routine analysis of drinking-water samples that are brought to the CSIR-WRI.

## 2. Materials and Methods

### 2.1. Study Design

This was a cross-sectional study using routinely collected data on drinking water samples from the Greater Accra region that were submitted for analysis at the CSIR-WRI microbiology laboratory between December 2021 and March 2022.

### 2.2. General Setting

Ghana is a West African country situated along the Gulf of Guinea, and has the Greater Accra region as its capital. The landscape is low lying, 20 m above sea level, with an average annual rainfall of 800 mm. The country has a population of about 30.8 million, and about 5.4 million live in Accra [20]. In Ghana, basic drinking water services are available to about 79% of the population, but this proportion includes those with access without considering the quality of the water [6,21]. For instance, in Accra, 98% of the population have access to basic drinking water; however, about 50% of the water tested at household levels is contaminated with *E. coli*. Currently, the main sources of drinking water available to residents are sachets, bottled water, tap water, boreholes, hand-dug wells and rain-harvested water [6]. Only 19% of the population have access to safely managed drinking water [6], and thus, access to reliable safe drinking water is still a key challenge in Ghana. The Ghana Standards Authority regulates the quality assurance standards of drinking water, and testing laboratories will refer to these standards, along with others from WHO, to perform the tests [12,13]. Currently, there is no requirement for testing drinking water sources for antimicrobial resistant bacteria.

### 2.3. Specific Setting

The CSIR–Water Research Institute, situated in Accra, Ghana, is one of the 13 institutes of the CSIR, the leading public research institution in Ghana. The institute generates and provides scientific information, strategies and services for the utilization and management of water resources in Ghana in support of the socioeconomic advancement of the country, especially in the agriculture, health, industry and energy sectors. The water-quality testing laboratory of the institute receives and tests drinking water samples from all over Ghana. However, due to the requirements of water needing to be tested within 24 h of collection, most water samples are from Accra and its surrounding communities because of the proximity of these communities to the laboratory. The laboratory receives an average of thirty samples per week from the Greater Accra region, including water samples from boreholes, hand-dug wells, surface water, tap water, sachets, and bottled drinking water. Samples are either brought to the laboratory for testing by water production companies or by individuals who want to know the quality of their drinking water. These water samples could either be untreated from a water source (i.e., boreholes, wells, and surface water) or treated. Water treatment methods used in Ghana include filtration, chlorination, reverse osmosis (RO) and ultraviolet (UV) light for the disinfection of drinking water.

### 2.4. Sample Collection

**Figure 1:**
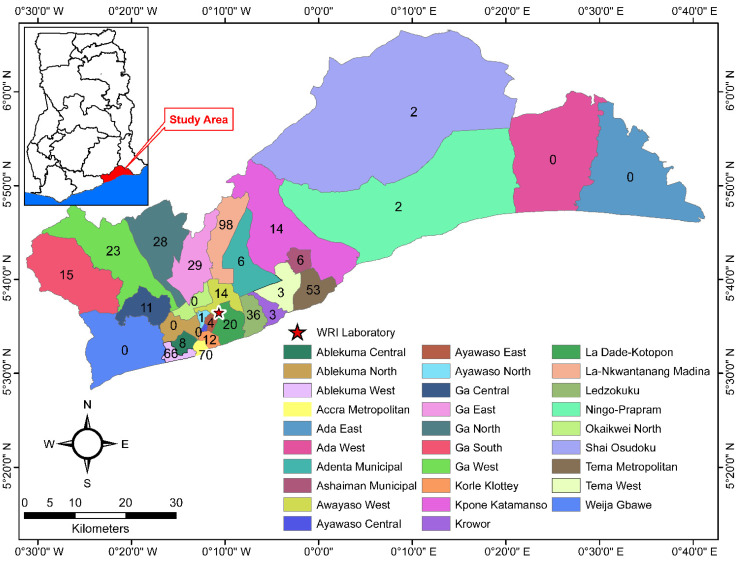
Map of Greater Accra region, Ghana, showing sample source locations and number of drinking water samples received and tested, December 2021–March 2022. WRI: CSIR–Water research Institute laboratory in Accra. Numbers indicate the number of samples received from each district.

A total of 524 potable water samples from the Greater Accra region were brought to the laboratory for analysis during the study period, December 2021 to March 2022. Samples were received from 23 out of the 29 districts in the region (Figure 1). The majority (n = 98, 18.7%) of the water samples were received from the La-Nkwatanang Madina district, followed by the Accra Metropolitan (n = 70), Ablekuma West (n = 66) and Tema Metropolitan (n = 53). Ayawaso North, Shai Osu doku and Ningo Prampram provided the least samples (n = 1, 2 and 2, respectively), with no samples from six districts (Figure 1). 

Out of the 524 samples received, 464 (88.5%) were from water and food processing companies needing to test samples before use or distribution, while 60 (11.5%) samples came from individuals testing for household use. Most samples were either packaged (sachet and bottled) water (n = 237, 45.2%), or treated water, whilst 69 (13.2%) samples were from untreated water sources (boreholes and wells) (Table 1).

Sachet and bottled water samples were submitted in their various packages, while tap, borehole and well water samples were submitted in 500 mL sterile sample bottles previously obtained from CSIR–Water Research Institute and labeled before they were submitted to the laboratory within 24 h for analysis. 

### 2.5. Sample Analysis

Water samples were analyzed according to procedures outlined in the Standard Methods for Examination of Water and Wastewater [22]. For total coliforms, *E. coli*, and *P. aeruginosa*, the membrane filtration technique was used. From each water sample, 100 mL was filtered and plated onto selective media, Chromocult Coliform Agar (Merck Millipore, Darmstadt, Germany) for isolation of TC and *E. coli*, and Cetrimide agar (Oxoid, Hampshire, UK) for isolation of *P. aeruginosa;* the plates were incubated at 37 °C ± 2 for 24 h. The pour plate technique was used to enumerate the THB count on nutrient agar (Oxoid, Hampshire, UK); the plates were then incubated at 37 °C ± 2 for 48 h. The microbial counts were reported as “colony forming units” (CFU) per 100 mL and per mL for THB. Presumptive colonies of *E. coli* and *P. aeruginosa* were randomly selected from each culture plate. These were then sub-cultured for purity and subsequent confirmation using Matrix-Assisted Laser Desorption Ionization–Time of Flight Spectrometry (MALDI-TOF MS) (Bruker MALDI Biotyper, Billerica, MA, USA). 

### 2.6. Antibiotic Susceptibility Testing

Pure colonies from each sample with *E. coli* and *P. aeruginosa* contamination were randomly selected for antibiotic susceptibility testing. Pure isolates of *E. coli*, and *P. aeruginosa* were subjected to antibiotic susceptibility testing using the Kirby–Bauer Disc Diffusion method on Mueller–Hinton agar, as recommended by Clinical Laboratory Standards Institute (CLSI) guidelines [23]. Zones of inhibition were measured in millimeters and recorded for each antibiotic. 

Antibiotics used for *E. coli* isolates included amoxicillin–clavulanate (20/10 µg), aztreonam (30 µg), ertapenem (10 µg), gentamicin (10 µg), chloramphenicol (20/10 µg), ciprofloxacin (5 µg), cefuroxime (30 µg), ceftriaxone (30 µg) and trimethoprim–sulphamethoxazole (1.25/23.75 µg) (Oxoid, Hampshire, UK). For *P. aeruginosa,* piperacillin–tazobactam (100/10µg), aztreonam (30 µg), gentamicin (10 µg) and ciprofloxacin (5 µg) (Oxoid, Hampshire, UK) were used.

### 2.7. Quality-Control Procedures

Negative controls were included by plating 100 mL of sterile distilled water and incubating these concurrently with the tested samples. This quality-control measure was performed to ensure bacterial loads recovered from samples were not influenced by laboratory conditions. Reference organisms *P. aeruginosa* ATCC 29213 and *E. coli* ATCC 25922 were used as controls in accordance with CLSI guidelines to ensure that the antibiotic disc diffusion process was consistent. The same sample analyses procedure was used each time.

### 2.8. Data Collection and Validation

Information on sample type, sample source location, bacterial loads and resistant profiles were entered into the CSIR-WRI electronic database. The variables of interest for this study were entered into an electronic Excel data file in a password-protected computer, which was kept in the laboratory. Two trained data assistants were responsible for data entry into the Excel file. To ensure data validation, all data entered in the Excel file were cross-checked and compared with the raw data contained in the laboratory electronic database by the PI.

### 2.9. Statistical Analysis

Data were exported into SPSS version 21 (Version 21.0. Armonk, NY: IBM Corp) for further analysis. Descriptive statistics, frequencies and proportions were computed to define the characteristics of drinking water samples brought for microbial analysis. Median counts for each bacterium (total coliform, total heterotrophic bacteria, *E. coli* and *P. aeruginosa*) were compared to WHO and Ghana Standard guidelines for drinking water of 0 CFU/100 mL for all bacteria analyzed and <500 CFU/mL for THB. Proportions that were within the WHO and Ghana Standard limits [13] were presented in text by sample type. Analysis of variance (ANOVA) and non-parametric tests (i.e., Kruskal–Wallis) were used to determine differences in the bacterial count from various sample types at 95% confidence intervals and presented in narrative text. Levels of significance were set at 5% (*p* < 0.05).

For Antimicrobial-Susceptibility Testing, the resistance profiles of the isolates were analyzed to determine if they were multidrug resistant (MDR) as per Magiorakos, 2012 [24]; that is, MDR was defined as resistance to three or more classes of antibiotics. Differences in proportions of antibiotic resistance between the different sample types and between treated and untreated samples were compared using the chi square test. Differences at the 5% level (*p* < 0.05) were regarded as significant. Water-source locations and MDR isolates were plotted by entering collected GPS coordinates in QGIS software (QGIS Development Team, Open Source Geospatial Foundation) to geographically describe the distribution of resistant isolates.

## 3. Results

Well water samples had the highest counts of TC (125.0; interquartile range (IQR), 40–275 cfu/100 mL) and *E. coli*, (45.0; IQR, 10–80 cfu/100 mL). Most packaged water (sachet and bottled) samples had no TC, *E. coli* or *P. aeruginosa* isolated; 98.4%, 100% and 92.6%, respectively for sachet water and 95.7%, 100% and 97.9% for bottled water. Most borehole and tap water samples were devoid of *E. coli* (94.8% and 84.6%, respectively) and *P. aeruginosa* (64.9% and 71.4%). THB counts were highest in well water (1493.5; IQR, 744–2790 cfu/mL), followed by boreholes (1196.0; IQR, 1–2808 cfu/mL) and tap water (676.0; IQR, 0–2808 cfu/mL). Overall counts varied significantly between different sample types for the bacteria tested (*p* < 0.05). However, there was no significant difference between sachet and bottled water samples for all four bacteria tested (*p* = 1.00). There was also no significant difference between tap and borehole water samples for all the bacteria tested (*p* > 0.05) (Table 2).

One hundred and fifteen *E. coli* isolates were tested against nine antibiotics and 202 *P. aeruginosa* isolates were tested against four antibiotics (Table 3). *E. coli* isolates from both treated (mainly tap) and untreated water (borehole and well) were most resistant to cefuroxime (91.5% and 81.8%, respectively), followed by trimethoprim–sulphamethoxazole (65.9% and 54.5%, respectively) and amoxicillin–clavulanate (54.9% and 45.5%, respectively). *E. coli* isolates from treated and untreated samples were least resistant to gentamicin (3.7% and 0.0%, respectively) and ertapenem (6.1% and 6.1%, respectively). There were no significant differences between the proportions of resistant *E. coli* isolates from treated and untreated water samples except for resistance to ciprofloxacin (*p* = 0.01). *P. aeruginosa* isolates were most resistant to aztreonam (52.1% in treated and 37.9% in untreated water), and least resistant to ciprofloxacin (4.9% in treated and 3.4% in untreated water). There were no significant differences between the proportion of resistant *P. aeruginosa* isolates in treated and untreated water samples tested (Table 3).

The proportion of MDR *E. coli* detected was 58.3%, whilst only 4.5% *P. aeruginosa* isolates were MDR. *E. coli* isolates from borehole water samples were more likely to be MDR, (72.7%; 8/11), followed by tap water (64.6%; 51/79) and well water (32.0%; 8/25) (*p* = 0.01). There was no significant difference (*p* = 0.83) between the proportion of MDR *P. aeruginosa* isolates from borehole (4/58), tap water (3/90) and sachet water (2/45) (Table 4).

**Figure 2:**
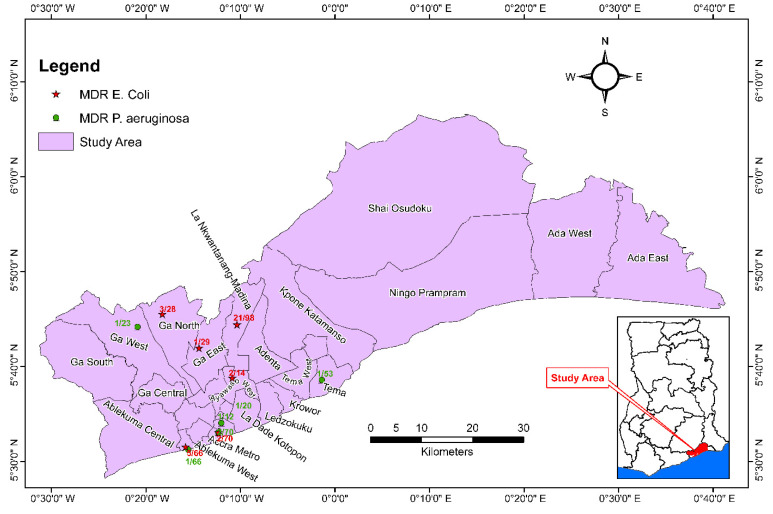
A spot map of Greater Accra region, Ghana showing source locations of MDR *E. coli* and *P. aeruginosa* isolates from water samples (December 2021–March 2022). MDR: Multidrug resistance.

Figure 2 shows the geographical distribution of samples where at least one MDR *E. coli* and/or one MDR *P. aeruginosa* isolate was detected. Of the 98 samples tested from the La-Nkwatanang Madina district, 21 had at least one MDR *E. coli* detected, followed by Ayawaso West (2/14), Ga North (3/28), Ga East (1/29), Ablekuma West (5/66) and Accra metropolitan (2/70). MDR *P. aeruginosa* was only isolated in 2/70 samples from Accra metropolitan and in one sample from Tema metropolitan (1/53), La Dade-kotopon (1/20), Korle Klottey (1/12), Ablekuma West (1/66) and Ga West municipal (1/23) (Figure 2). 

For the number of isolates tested and the number of MDR bacteria per each district, see Supplementary Table S1.

## 4. Discussion

This study is the first to examine antibiotic resistance patterns of both *E. coli* and *P. aeruginosa* isolates in different drinking water sources from the Greater Accra region in Ghana. There were three major findings.

First, the majority of packaged water samples had no bacterial contamination, or they were within limits of the WHO and Ghana Standards guidelines for safe drinking water [12,13]. This confirms the efficiency of treatment methods being used by these companies to reduce bacteria. Most borehole and tap water samples did not have *E. coli* counts, and over 50% of samples had no detectable *P. aeruginosa.* These observations are similar to other reports in Ghana [21,25,26]. However, all well water samples were contaminated and had high counts of *E. coli*, which corroborates with other findings in Ghana [27] and Cameroon [28]. The high counts of *E. coli* could probably be attributed to recent fecal contamination of the water source. 

Second, although the majority of the water samples in this study had no *E. coli* or *P. aeruginosa* counts, over half of the *E. coli* isolates tested were MDR. Overall, *E. coli* isolates were most resistant to cefuroxime (88.7%), trimethoprim–sulphamethoxazole (62.6%) and amoxicillin–clavulanate (52.2%), while *P. aeruginosa* isolates were most resistant to aztreonam (48%). Bacterial isolates were most susceptible to gentamicin, ertapenem and ciprofloxacin. These findings are in line with previous reports of resistant *E. coli* isolates in some water sources in the northern region of Ghana, which were 96.2% resistant to cefuroxime and 86.5% susceptible to ciprofloxacin [16]. 

A review of antibiotic resistance in Ghana (40% in Accra) with data mainly from human isolates (85%) reported a similar high resistance (over 50%) in *E. coli* isolates to trimethoprim–sulphamethoxazole, amoxicillin–clavulanate and cefuroxime with relatively lower resistance to gentamicin and ciprofloxacin [19]. A recent study also showed that *E. coli* and *P. aeruginosa* isolates from a waste treatment plant and a receiving stream in Accra had higher resistance to amoxicillin–clavulanate, cefuroxime and aztreonam [29]. It is therefore possible that the results seen in drinking water sources may be due to contamination from untreated human and animal waste discharged into the environment. Thus, these contaminated water sources are carriers of ARB, contributing to the dissemination of AMR. It is important to note that cefuroxime and amoxicillin–clavulanate are among the most prescribed antibiotics in human health in Ghana [30,31]; the excessive use of these antibiotics and inappropriate disposal may be contributing to resistance by promoting the development of resistance in bacteria under selective pressure [32]. 

Third, there was no significant difference between the proportion of resistant isolates from treated (mainly tap water) and untreated (borehole and well) water sources to the various antibiotics tested. This may be because there is usually a deterioration in tap-water quality along distribution lines [33] and also because the formation of biofilms in pipe networks increases resistance in microorganisms, as these biofilms protect them from the effects of antimicrobial agents [34,35]. Furthermore, conventional water treatment methods at water treatment plants are generally unable to adequately remove ARB from water [32]. In Ghana, chlorination is the main treatment method for tap water. However, several studies have shown that ARB are tolerant to chlorination, and this process may actually promote the development of antimicrobial-resistant genes [36,37]. Research has shown that a combination of UV/chlorine is more efficient for removing ARB than the individual UV or chlorination process alone [32,38]. 

Although the La-Nkwatanang Madina district recorded the highest number of MDR samples and isolates, this observation was mainly due to the large number of samples analyzed from this district.

The findings from this study are of public health importance, as they demonstrate the vital role that safe drinking water plays in protecting human and animal health. Both humans and animals are likely to benefit from safe drinking water through reduced incidence of water-borne diseases and reduced acquisition of antibiotic resistance, which is of benefit to “One Health” [39]. This study also shows the potential importance of drinking-water sources, including borehole and tap water, in the spread of AMR in Ghana. ARB and ARGs can be transferred from one community to another through water distribution systems. Thus, people may be exposed to ARB in contaminated water, resulting in infections that are difficult to treat. 

The strengths of this study are that: a large sample size was used compared to previous studies; all water samples were analyzed within 24 h of submission to the laboratory by a senior laboratory technician to prevent bacterial overgrowth; antibiotic susceptibility testing was performed according to international standards, with quality-control measures in place [22,23]; the antibiotics tested included those listed by the WHO as being critically important in human medicine [40]; and the subject matter addresses an identified national operational research priority for tackling AMR. We also adhered to STROBE (strengthening the reporting of observational studies in epidemiology) guidelines for the conduct and reporting of this study [41].

However, we acknowledge some limitations of this study. We only targeted samples submitted to the laboratory for analysis and were unable to compare MDR prevalence across the region due to a low number of samples being sent from some districts. Testing additional samples from those areas and possibly a nationwide surveillance could be considered in future research. We were unable to assess seasonal differences due to the short study period, and this will be valuable to consider in future. Additionally, antibiotic resistance testing was limited to only two organisms. However, this was a deliberate choice, as the selected bacteria are known to be indicators of drinking-water quality, and they serve as proxies for AMR surveillance in water. We would therefore recommend that future studies consider more organisms for evaluation.

There are some important policy and practice implications. First, the low level of antibiotic resistance to ciprofloxacin, gentamicin, ertapenem, ceftriaxone and chloramphenicol is encouraging, as these antibiotics are often used as first- and second-line treatments in humans. However, the similarities in resistance patterns of *E. coli* isolates to those reported in human and animal isolates [19] is alarming. This could mean a possible transfer of ARB from humans to the environment and drinking-water sources. That said, the majority of water samples were free of *E. coli* and *P. aeruginosa* contamination. Thus, less contaminated water means less ARB spread. Findings from this study could serve as a justification for the continuous surveillance of drinking water sources, as well as advocacy for including AST as part of the parameters for routine analysis of water samples brought to the CSIR–Water Research Institute. This would be a potential benefit of the One Health approach in tackling AMR. 

Furthermore, the presence of MDR isolates in both tap and groundwater samples suggests that there may be contamination in the water-distribution lines and seepage of inadequately treated sewage into groundwater. We would like to call for immediate measures that would include informing and engaging stakeholders to improve their awareness of the presence of these pathogens in drinking-water sources and educating communities on additional cost-effective water-treatment methods; for example, boiling water before household use. Individuals should also be encouraged to submit water for testing, as a robust surveillance system would encapsulate records from both households and water producing companies. 

In the long term, policy considerations about increasing the number of sewage treatment plants in Accra and enforce legislation that prevents indiscriminate discharge of household sewage into the environment and water bodies should be considered. Currently, Accra has four main sewage treatment plants located at the Ga West, Ayawaso West, Ledzokuku and Accra Metropolitan districts. The sewage management plan covers only 30% of the waste generated, and only 15% of the total land area is connected to the sewer system [7,42]. Regular repairs, maintenance and replacement of existing water-distribution pipelines should also be carried out to prevent contamination through burst pipes, as this would maintain the quality of water reaching households. This is imperative if Ghana is to achieve the SDG goal of “achieving safe water for all” by the year 2030.

Further work should also be carried out to determine the resistance genes, virulence factors and other characteristics present in antibiotic resistant bacteria from drinking-water sources.

## 5. Conclusions

In conclusion, most of the water samples analyzed in this study were free of *E. coli* and *P. aeruginosa*. However, isolates with similar resistance patterns to isolates from human and animal studies were observed. This study has confirmed the presence of ARB in drinking water sources (tap, borehole and well) in Ghana and provides evidence for the need for routine monitoring and surveillance. These findings highlight the important role drinking water can play in the dissemination of AMR. Immediate measures must be taken in order to enhance awareness of stakeholders and communities regarding water-treatment methods, as well as the negative impact of indiscriminate sewage disposal in the environment.

## Figures and Tables

**Table 1:** Characteristics of water samples brought to the Water Research Institute for microbial water quality analysis from suburbs within Greater Accra region, Ghana (December 2021–March 2022).

Characteristics		N	%
Total		524	100
Sample provider	Individual	60	11.5
	Water or food processing company	464	88.5
Sample type	Sachet	190	36.3
	Bottled	47	9.0
	Tap water	196	37.4
	Borehole water	77	14.7
	Well water	14	2.7
Treatment methods used	Untreated	69	13.2
	Chlorination only	171	32.6
	Chlorination, Filtration	7	1.3
	Chlorination, Filtration, Ultrafiltration, UV	20	3.8
	Filtration, UV	11	2.1
	RO, Filtration	14	2.7
	Sand, Carbon, RO, Filtration, UV	232	44.3

UV: ultraviolet, RO: reverse osmosis, Sand: sand filtration, Carbon: carbon filtration.

**Table 2:** Bacterial load (cfu/100 mL) of various drinking water samples from Greater Accra region, Ghana (December 2021–March 2022).

		Type of Bacteria							
Sample Type	No. Samples	Total Coliforms		*E. coli*		*P. aeruginosa*		THB	
		Median	IQR	Median	IQR	Median	IQR	Median	IQR
Sachet	190	0.0	0–0	0.0	0–0	0.0	0–0	1.0	0–28
Bottle	47	0.0	0–0	0.0	0–0	0.0	0–0	0.0	0–14
Borehole	77	5.0	0–111	0.0	0–0	0.0	0–21	1196.0	1–2808
Tap	196	13.0	0–465	0.0	0–0	0.0	0–10	676.0	0–2808
Well	14	125.0	40–275	45.0	10–80	0.0	0–36	1493.5	744–2790

cfu: colony forming units; IQR: interquartile range. THB: total heterotrophic bacteria.

**Table 3:** Number and proportion (%) of antibiotic resistant *Escherichia coli* and *Pseudomonas aeruginosa* isolates in drinking water samples from Greater Accra region, Ghana (December 2021–March 2022).

	*E. coli*					*P. aeruginosa*				
Antibiotics (μg)	Treated (N = 82)		Untreated (N = 33)		*p*	Treated (N = 144)		Untreated (N = 58)		*p*
	n	%	n	%		n	%	n	%	
Amoxicillin–clavulanate (20/10 µg)	45	54.9	15	45.5	0.36	-	-	-	-	-
Piperacillin–tazobactam (100/10 µg)	-	-	-	-	-	14	9.7	5	8.6	0.8
Gentamicin (10 µg)	3	3.7	0	0.0	0.26	16	11.1	7	12.1	0.84
Ciprofloxacin (5 µg)	14	17.1	0	0.0	0.01	7	4.9	2	3.4	0.66
Aztreonam (30 µg)	13	15.9	8	24.2	0.29	75	52.1	22	37.9	0.06
Cefuroxime (30 µg)	75	91.5	27	81.8	0.14	-	-	-	-	-
Ertapenem (10 µg)	5	6.1	2	6.1	0.99	-	-	-	-	-
Trimethoprim–sulfamethoxazole (1.25/23.75 µg)	54	65.9	18	54.5	0.25	-	-	-	-	-
Chloramphenicol (30 µg)	28	34.1	8	24.2	0.3	-	-	-	-	-
Ceftriaxone (30 µg)	28	34.1	7	21.2	0.17	-	-	-	-	-

N: number of total isolates; n: number of resistant isolates; -: antibiotic was not tested against isolates.

**Table 4:** Number and proportion of multidrug resistance in *E. coli* and *P. aeruginosa* isolates in drinking-water samples tested from Greater Accra region, Ghana (December 2021–March 2022).

	*E. coli*		*P. aeruginosa*	
	n (N)	%	n (N)	%
Total Isolates	67 (115)	58.3	9 (202)	4.5
Sachet	-	-	2 (45)	4.4
Bottled	-	-	0 (3)	0.0
Tap	51 (79)	64.6	3 (90)	3.3
Borehole	8 (11)	72.7	4 (58)	6.9
Well	8 (25)	32.0	0 (6)	0.0

n: number of multidrug resistant isolates; N: number of isolates tested; -: no bacteria was isolated for that sample.

## Data Availability

The metadata record of the data used in this paper is available at https://doi.org/10.6084/m9.figshare.20459403 (accessed on 10 August 22) and is available under a CC BY 4.0 license.

## Supplementary Materials

The following supporting information can be downloaded at: https://www.mdpi.com/article/10.3390/ijerph191912300/s1, Table S1: Supplementary table showing various districts and number of multidrug resistant isolates.
