# Supplementary material for: Antibiotic-Resistant Bacteria in Drinking Water from the Greater Accra Region, Ghana: A Cross-Sectional Study, December 2021–March 2022
Source: Int J Environ Res Public Health. 2022 Sep 28;19(19):12300. doi: 10.3390/ijerph191912300 (PMC9566567; doi:10.3390/ijerph191912300)
Supplement: Supplementary file 1 [file ijerph-19-12300-s001.zip › Supplementary Table S1.pdf]

Table S1. Supplementary table showing various districts and number of multidrug resistant isolates

District	Total No. Samples	Water type					No. isolates tested			
		Sachet	Bottled	Tap	Borehole	Well	*E. coli*		*P. aeruginosa*	
							Total	MDR	Total	MDR
La-Nkwatanang Madina	98	4	0	77	4	13	65	38	6	0
Accra Metropolitan	70	26	4	22	18	0	8	5	18	2
Ablekuma West	66	12	0	53	1	0	19	12	43	1
Tema Metropolitan	53	23	4	23	3	0	0	0	29	1
Ledzokuku	36	10	5	12	9	0	0	0	13	0
Ga East	29	18	7	0	3	1	6	0	10	0
Ga North	28	14	2	2	10	0	10	9	10	0
Ga West	23	14	4	1	4	0	0	0	4	1
La Dade-Kotopon	20	8	5	1	6	0	0	0	2	2
Ga South	15	12	0	0	3	0	0	0	13	0
Ayawaso West	14	10	0	1	3	0	7	3	23	0
Kpone Katamanso	14	2	12	0	0	0	0	0	6	0
Korle Kottley	12	1	1	1	9	0	0	0	5	2
Ga Central	11	11	0	0	0	0	0	0	0	0
Ablekuma Central	8	5	1	1	1	0	0	0	5	0
Adenta Municipal	6	5	0	0	1	0	0	0	0	0
Ashaiman Municipal	6	6	0	0	0	0	0	0	5	0
Ayawaso East	4	3	0	0	1	0	0	0	1	0
Dangme West	3	3	0	0	0	0	0	0	9	0
Krowor	3	1	0	2	0	0	0	0	0	0
Ningo/Prampram	2	1	1	0	0	0	0	0	0	0
Shai Osudoku	2	0	0	0	2	0	0	0	0	0
Ayawaso North	1	1	0	0	0	0	0	0	0	0
Ayawaso Central	-	-	-	-	-	-	-	-	-	-
Ablekuma North	-	-	-	-	-	-	-	-	-	-
Ada East	-	-	-	-	-	-	-	-	-	-
Ada West	-	-	-	-	-	-	-	-	-	-
Okaikwei North	-	-	-	-	-	-	-	-	-	-
